# Perfluorocyclobutyl Aryl Ether-Based ABC Amphiphilic Triblock Copolymer

**DOI:** 10.1038/srep39504

**Published:** 2016-12-21

**Authors:** Binbin Xu, Wenqiang Yao, Yongjun Li, Sen Zhang, Xiaoyu Huang

**Affiliations:** 1 Key Laboratory of Synthetic and Self-Assembly Chemistry for Organic Functional Molecules, Shanghai Institute of Organic Chemistry, Chinese Academy of Sciences, 345 Lingling Road, Shanghai 200032, People’s Republic of China

## Abstract

A series of fluorine-containing amphiphilic ABC triblock copolymers comprising hydrophilic poly(ethylene glycol) (PEG) and poly(methacrylic acid) (PMAA), and hydrophobic poly(*p*-(2-(4-biphenyl)perfluorocyclobutoxy)phenyl methacrylate) (PBPFCBPMA) segments were synthesized by successive atom transfer radical polymerization (ATRP). First, PEG-Br macroinitiators bearing one terminal ATRP initiating group were prepared by chain-end modification of monohydroxy-terminated PEG via esterification reaction. PEG-*b*-PBPFCBPMA-Br diblock copolymers were then synthesized via ATRP of BPFCBPMA monomer initiated by PEG-Br macroinitiator. ATRP polymerization of *tert*-butyl methacrylate (*t*BMA) was directly initiated by PEG-*b*-PBPFCBPMA-Br to provide PEG-*b*-PBPFCBPMA-*b*-P*t*BMA triblock copolymers with relatively narrow molecular weight distributions (*M*_w_/*M*_n_ ≤ 1.43). The pendant *tert*-butyoxycarbonyls were hydrolyzed to carboxyls in acidic environment without affecting other functional groups for affording PEG-*b*-PBPFCBPMA-*b*-PMAA amphiphilic triblock copolymers. The critical micelle concentrations (*cmc*) were determined by fluorescence spectroscopy using *N*-phenyl-1-naphthylamine as probe and the self-assembly behavior in aqueous media were investigated by transmission electron microscopy. Large compound micelles and bowl-shaped micelles were formed in neutral aqueous solution. Interestingly, large compound micelles formed by triblock copolymers can separately or simultaneously encapsulate hydrophilic Rhodamine 6G and hydrophobic pyrene agents.

Owing to the unique properties^1^,^2^,^3^,^4^,^5^ (high thermostability, high insulating ability, excellent chemical inertness, aging and weather resistances, low refractive index, and low surface energy) originating from low polarizability, strong electronegativity, small van der Waals radius of F atom, and strong C-F bond, fluoropolymers are extensively employed as a class of high performance materials in recent years^6^,^7^,^8^,^9^. However, along with the high crystallinity and thermal and chemical resistances, most fluoropolymers are difficult to dissolve or melt so that industrial manufacturing would be costly and technically difficult. These issues are not easy to be solved, which hinder the development and application of fluoropolymers.

Perfluorocyclobutyl (PFCB) aryl ether-based polymer is one kind of partially fluorinated polymers which was developed by Babb *et al*.^10^,^11^ of Dow Chemical in early 1990s. Compared with other commercial fluoropolymers, PFCB aryl ether-based polymer has analogous properties. Furthermore, due to the entire amorphism and good solubility in common organic solvents, PFCB aryl ether-based polymer has better processability^12^,^13^, which have greatly simplified some manufacturing procedures. Meanwhile, because of the desirable properties such as low dielectric constant, low moisture absorption, good oxidative resistance, good thermal stability, low birefringence, and excellent optical transparency^14^,^15^,^16^, PFCB aryl ether-based polymers have been widely explored as photonics, atomic oxygen resistant coatings, hybrid composites, polymer light-emitting diodes, liquid crystals, and proton exchange membranes for fuel cells^17^,^18^,^19^,^20^,^21^,^22^.

Recently, fluorinated polymethacrylates which exhibit hybrid properties of polymethacrylate and fluoropolymer have been extensively used in various areas^4^. Fluorinated polymethacrylates are usually obtained via the strategy of homopolymerization or copolymerization of fluorinated methacrylates with other methacrylic monomers. The cost-effectiveness, easy processability, and good reactivity of fluorinated methacrylates make the products more attractive^23^. In addition, because of the remarkably low surface energies, stain resistance, low friction coefficients, and good optical properties, such fluorinated polymethacrylates may be attractive for the preparation of high performance coating surfaces, medical materials, functional membranes, optical devices, and fibers^24^,^25^,^26^,^27^.

Great attention has been paid to the synthesis of various fluoropolymers, and a large number of fluoropolymers with controlled molecular weights and narrow molecular weight distributions have emerged with the rapid development of polymerization techniques^28^,^29^. Inspired by the excellent properties of PFCB aryl ether-based polymers and fluorinated methacrylates, our group has reported a new fluorinated methacrylate monomer of *p*-(2-(4-biphenyl)perfluorocyclobutoxy)phenyl methacrylate (BPFCBPMA) containing PFCB aryl ether backbone^23^. By using BPFCBPMA and isobutylene as starting material, we have synthesized PBPFCBPMA-*b*-PIB-*b*-PBPFCBPMA amphiphilic ABA triblock copolymers by tandem living carbocationic polymerization and ATRP via the site transformation strategy. For the combination of hydrophobicity and lipophobicity in fluorinated polymers, PBPFCBPMA-*b*-PIB-*b*-PBPFCBPMA triblock copolymers exhibited interesting self-assembly characteristics. Previous studies showed that amphiphilic copolymers with different chain architecture could self-assemble into diverse nanostructures^30^,^31^,^32^. Therefore, synthesis of PFCB aryl ether-containing amphiphilic copolymers bearing hydrophilic and lipophobic segments would not only yield the fluoropolymers with different architectures, but deepen our understanding on the structure-property relationship of PFCB aryl ether-based polymethacrylates.

Herein, on the basis of semi-fluorinated BPFCBPMA monomer, we report the synthesis of fluorine-containing amphiphilic ABC triblock copolymer of PEG-*b*-PBPFCBPMA-*b*-PMAA with hydrophilic poly(ethylene glycol) (PEG) and poly(methacrylic acid) (PMAA) blocks and hydrophobic poly(*p*-(2-(4-biphenyl)perfluorocyclobutoxy)phenyl methacrylate) (PBPFCBPMA) segment as shown in Fig. 1. We take advantage of the versatility of atom transfer radical polymerization (ATRP)^33^,^34^,^35^,^36^ to synthesize PEG-*b*-PBPFCBPMA-Br diblock copolymers and PEG-*b*-PBPFCBPMA-*b*-P*t*BMA triblock copolymers using bromine- functionalized PEG as macroinitiators. The amphiphilicity of triblock copolymers could be further tuned by selective acidic hydrolysis of hydrophobic P*t*BMA segment into hydrophilic poly(methacrylic acid) (PMAA) block to afford PEG-*b*-PBPFCBPMA-*b*-PMAA triblock copolymers while PEG and PBPFCBPMA segments kept inert. It was found that PEG-*b*-PBPFCBPMA-*b*-PMAA amphiphilic triblock copolymers bearing hydrophilic PEG, PMAA, and lipophobic PBPFCBPMA segments could self-assemble into large compound micelles in aqueous solution. The encapsulating capacities of large compound micelles were examined by using hydrophilic Rhodamine 6G (R6G) and hydrophobic pyrene as model loading agents. The results showed large compound micelles with a special multi-compartment interior structure can sequester pyrene and R6G in hydrophobic and hydrophilic nanodomains, respectively, which is different from common spherical micelles just able to encapsulate hydrophobic agents.

## Results and Discussion

### Preparation of PEG-Br macroinitiator

In the current case, a fluorine-containing amphiphilic ABC triblock copolymer comprising a hydrophobic semi-fluorinated polymethacrylate segment (PBPFCBPMA) was first designed to be formed in a well-defined way via successive ATRP which becomes a powerful tool for the preparation of well-defined block polymers. For the selection of hydrophilic block, PEG is undoubtedly the first choice because it is a kind of water-soluble, uncharged, and nontoxic polymer^37^. Due to its hydrophilicity and biocompatibility, PEG not only increases the solubility of copolymers in aqueous media, but improves the circulation times *in vivo* when bound to other molecules^38^,^39^,^40^. Therefore, PEG has been extensively investigated for nanotechnology and biomedicine applications^41^,^42^,^43^,^44^. Obviously, the incorporation of PEG into fluoropolymers would endow fluoropolymers with a series of additional properties.

For the construction of amphiphilic block copolymer containing PEG segment, PEG-based macroinitiatior or chain transfer agent is usually employed for the subsequent reversible-deactivation radical polymerization (RDRP) including ATRP and reversible addition-fragmentation chain transfer (RAFT) polymerization^45^,^46^. In the current case, two methacrylate monomers of BPFCBPMA and *t*BMA, which are suitable for ATRP, are used for the construction of PEG-based amphiphilic ABC triblock copolymer. Thus, halogen-containing PEG-Br macroinitiator was first prepared by chain-end modification of monohydroxy-terminated PEG-OH via esterification^47^. Two PEG-OHs with different molecular weights were esterified with 2-bromopropionyl bromide using DMAP and TEA as catalysts, giving two PEG-Br **1** macroinitiators as summarized in Table 1. Both macroinitiators showed unimodal and symmetrical elution peaks with narrow molecular weight distributions (*M*_w_/*M*_n_ ≤ 1.13) in GPC curves, which meant the polymeric skeleton was kept during esterification.

**Figure 2:**
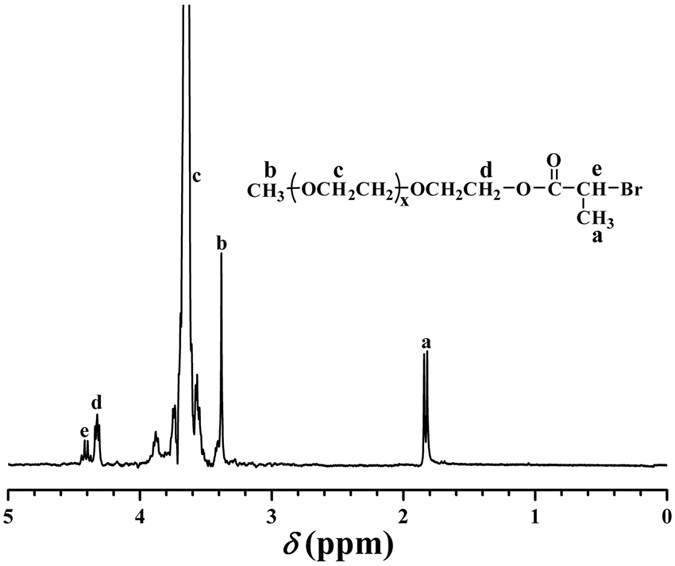
^1^H NMR spectrum of PEG-Br **1** macroinitiator in CDCl_3_.

The successful introduction of ATRP initiating groups was demonstrated by FT-IR and ^1^H NMR. A new peak appeared at 1741 cm^−1^ in FT-IR spectrum after esterification compared to that before esterification, which is attributed to the newly incorporated ester group. After the esterification, two new resonance signals including 1 proton and 3 protons of -COC*H*(C*H*_3_)Br initiating group are located at 4.42 ppm (peak “e”) and 1.84 ppm (peak “a”) in ^1^H NMR spectrum (Fig. 2), respectively. Typical resonance signals of PEG chain appeared at 3.38 ppm (peak “b”, OC*H*_3_) and 3.65 ppm (peak “c”, OC*H*_2_C*H*_2_). Moreover, the integration area ratio of peak “a” (COCH(C*H*_3_)Br) and peak “b” (OC*H*_3_) to peak “e” (COC*H*(CH_3_)Br) is almost 3:3:1, which verified the complete esterification of terminal hydroxyl of PEG.

GPC analysis using linear poly(ethylene glycol) standards was utilized to provide the relative molecular weights (*M*_n,GPC_) of PEG-Br **1** macroinitiators and the values are as listed in Table 1. It should be noted that *M*_n,GPC_ is very close to the theoretical molecular weight so that we can confirm the successful preparation of PEG-Br macroinitiator bearing terminal -COCH(CH_3_)Br initiating group, which can directly initiate ATRP of another monomer for constructing block copolymers.

### Synthesis of PEG-*b*-PBPFCBPMA-Br diblock copolymer

**Figure 3:**
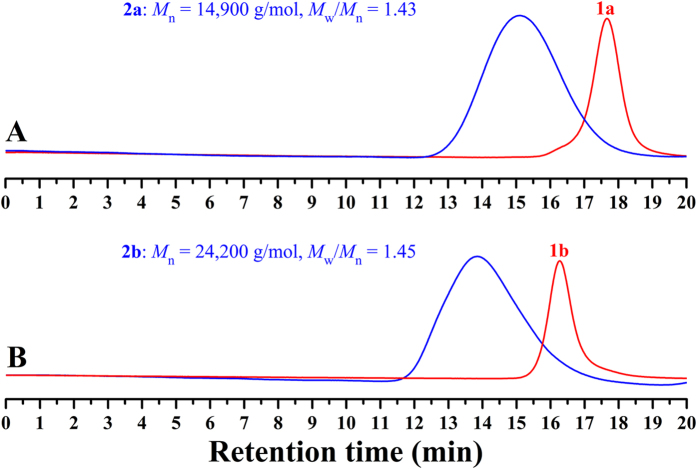
GPC curves of PEG-Br 1 macroinitiator and PEG-*b*-PBPFCBPMA-Br **2** diblock copolymer in THF.

On the basis of as-prepared PEG-Br macroinitiator, hydrophobic fluorine- containing PBPFCBPMA block was built via ATRP of semi-fluorinated BPFCBPMA methacrylate monomer. ATRP of BPFCBPMA was initiated by terminal -COCH(CH_3_)Br initiating group of PEG-Br **1** macroinitiator in 2-butanone at 70 °C using CuBr/PMDETA as catalytic system^23^. Two PEG-*b*-PBPFCBPMA-Br **2** diblock copolymers were obtained by using different PEG-Br **1** macroinitiator and different feeding ratio and the experimental details as well as the molecular weights and molecular weight distributions of PEG-*b*-PBPFCBPMA-Br diblock copolymers are summarized in Table 2. Both PEG-*b*-PBPFCBPMA-Br **2** diblock copolymers showed unimodal and symmetrical elution peaks with relatively narrow molecular weight distributions (*M*_w_/*M*_n_ ≤ 1.45) in GPC curves as shown in Fig. 3, and the molecular weights of both diblock copolymers were much higher than those of corresponding macroinitiators, which indicated the successful performance of ATRP of BPFCBPMA methacrylate monomer. Copolymer **2** was also washed with methanol, the precipitator for copolymer **2** and the solvent for PEG-Br **1**, and no trace of PEG-Br **1** was detected in the filtrate using GPC and ^1^H NMR, this indicating the complete initiation.

**Figure 4:**
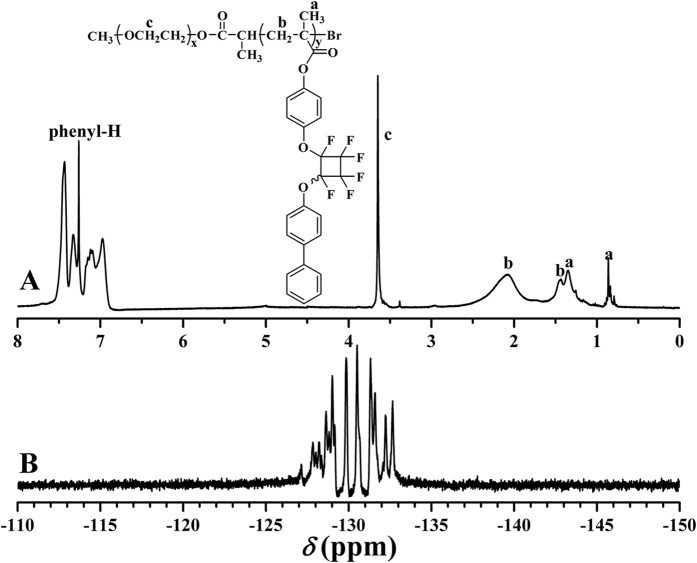
^1^H (**A**) and ^19^F (**B**) NMR spectra of PEG-*b*-PBPFCBPMA-Br **2** diblock copolymer in CDCl_3_.

The chemical structure of the resultant after ATRP of BPFCBPMA monomer was examined by FT-IR, ^1^H NMR, ^13^C NMR, and ^19^F NMR. Two new peaks corresponding to PFCB and aryl rings appeared at 962 and 1502 cm^−1^ in FT-IR spectrum after ATRP of BPFCBPMA, which affirmed the incorporation of PFCB aryl ether moieties. Figure 4A shows ^1^H NMR spectrum after ATRP of BPFCBPMA and all proton resonance signals of both EG and BPFCBPMA repeated units appeared in the spectrum. The proton resonance signal of double bond (5.00–7.00 ppm) disappeared after ATRP and the signal of 2 protons of -C*H*_2_C- in the polymethacrylate backbone of PBPFCBPMA segment appeared at 1.44 and 2.08 ppm (peak “b”), which clearly confirmed the successful polymerization of BPFCBPMA monomer. The strong peak at 3.65 ppm (peak “c”) belonged to 4 protons of -OC*H*_2_C*H*_2_- in EG repeated unit. The proton resonance signals located between 6.98 ppm and 7.44 ppm combining with the signals at 105.0, 119.5, 138.3, 146.3, and 152.9 ppm in ^13^C NMR spectrum and −127.0~−132.8 ppm in ^19^F NMR spectrum (Fig. 4B) also witnessed the existence of pendent PFCB aryl ether groups after ATRP of BPFCBPMA, indicative of successful formation of PBPFCBPMA block.

As we have mentioned in the experiment part that our GPC system was calibrated with linear poly(methyl methacrylate) standards, therefore, the molecular weights after ATRP of BPFCBPMA measured by conventional GPC (Table 2) may not reflect the “actual” value. In the present work, the number of BPFCBPMA repeated unit (y) could be estimated according to Eq. 1 (*S*_c_ represents the integration area of 4 protons of -OC*H*_2_C*H*_2_- at 3.65 ppm, and *S*_a_ and *S*_b_ represent the integration area of 3 and 2 protons of -C*H*_2_CC*H*_3_- at high field in Fig. 4A; x is the number of EG repeated unit obtained from the molecular weight of PEG-OH). Meanwhile, the “absolute” molecular weight (*M*_n,NMR_) of PEG-*b*-PBPFCBPMA-Br **2** diblock copolymer was calculated according to Eq. 2 (44 and 508 are the molecular weights of EG and BPFCBPMA repeated unit, respectively) and the results are summarized in Table 2. It can be clearly seen from Table that *M*_n,NMR_ is much higher than that obtained from GPC (*M*_n,GPC_).


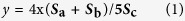



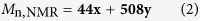


Thus, all above-mentioned results demonstrated the successful synthesis of PEG-*b*-PBPFCBPMA-Br **2** diblock copolymers comprising hydrophilic PEG and hydrophobic fluorine-containing PBPFCBPMA segments.

### Synthesis of PEG-*b*-PBPFCBPMA-*b*-P*t*BMA triblock copolymer

In the current work, *t*BMA monomer was selected for the construction of ABC amphiphilic triblock copolymer because hydrophobic P*t*BMA segment could be easily transformed into hydrophilic PMAA segment via selective acidic hydrolysis^48^,^49^,^50^. Therefore, based on as-prepared PEG-*b*-PBPFCBPMA-Br **2** diblock copolymer, ABC triblock copolymer was then constructed via ATRP of *t*BMA in 2-butanone at 70 °C using CuBr/PMDETA as catalytic system as summarized in Table 3. It can be seen from Table 3 that the molecular weight of triblock copolymer could be tuned by varying the feeding ratio.

**Figure 5:**
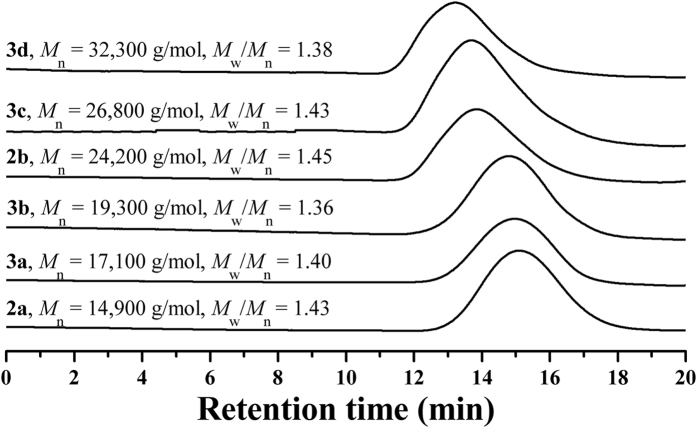
GPC curves of PEG-*b*-PBPFCBPMA-Br **2** diblock copolymer and PEG-*b*-PBPFCBPMA-*b*-P*t*BMA **3** triblock copolymer in THF.

Figure 5 shows GPC traces of the resultants after ATRP of *t*BMA, i.e. PEG-*b*-PBPFCBPMA-*b*-P*t*BMA **3** triblock copolymers. It was found that all triblock copolymers showed unimodal and symmetrical elution peaks with relatively narrow molecular weight distributions (*M*_w_/*M*_n_ ≤ 1.43), which illustrated that intermolecular coupling could be neglected^51^. In addition, the molecular weights of all copolymers were higher than that of the corresponding macroinitiator, which verified that PEG-*b*-PBPFCBPMA-Br **2** could act as macroinitiator to initiate ATRP of *t*BMA.

**Figure 6:**
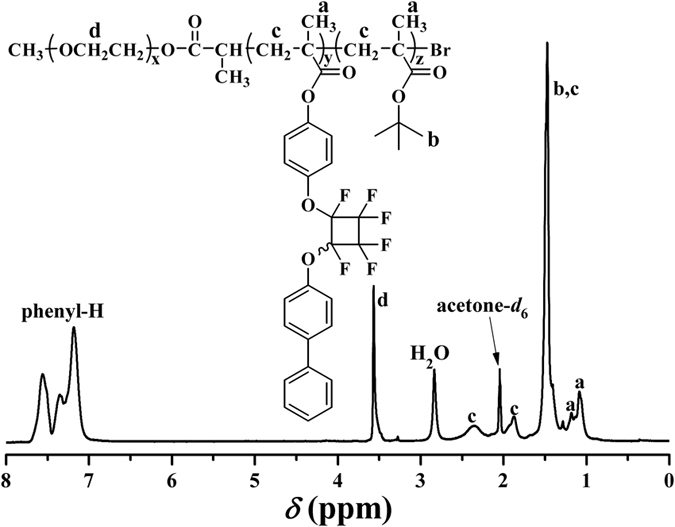
^1^H NMR spectrum of PEG-*b*-PBPFCBPMA-*b*-P*t*BMA 3 triblock copolymer in acetone-*d*_6_.

PEG-*b*-PBPFCBPMA-*b*-P*t*BMA **3** triblock copolymer was characterized by ^1^H NMR and ^13^C NMR, respectively. Figure 6 shows ^1^H NMR spectrum of the triblock copolymer and all the corresponding proton resonance signals originating from PEG, PBPFCBPMA, and P*t*BMA segments appeared in the spectrum. In particular, the resonance signal of double bond was absent in the spectrum and the strong peak at 1.47 ppm belonged to 9 protons of *tert-*butyls of P*t*BMA block, which witnessed the existence of P*t*BMA segment, i.e. successful ATRP of *t*BMA monomer. The typical proton resonance signal of PEG segment was located at 3.58 ppm (peak “d”) and the multiplets ranging from 7.16 ppm to 7.58 ppm were attributed to 13 protons of phenyl of PBPFCBPMA segment. Moreover, two new resonance signals assigned to the carbons of *tert-*butyl appeared at 27.9 (C(*C*H_3_)_3_) and 80.5 (*C*(CH_3_)_3_) ppm in ^13^C NMR spectrum after ATRP of *t*BMA^52^, which also indicated the formation of P*t*BMA block.

Similarly, the number of *t*BMA repeated unit (z) was evaluated according to Eq. 3 (*S*_a_, *S*_d_, and *S*_phenyl_ represent the integration area of 3 protons of CH_2_CC*H*_3_ at 1.09 and 1.18 ppm, 4 protons of -OC*H*_2_C*H*_2_- at 3.58 ppm, and 13 protons of phenyl ranging from 7.16 ppm to 7.56 ppm in Fig. 6, respectively; x is the number of EG repeated unit obtained from the molecular weight of PEG-OH) and the “absolute” molecular weight (*M*_n,NMR_) of PEG-*b*-PBPFCBPMA-*b*-P*t*BMA **3** triblock copolymer was calculated according to Eq. 4 (142 is the molecular weight of *t*BMA repeated unit) as summarized in Table 3. Indeed, *M*_n,NMR_ is much higher than that obtained from GPC.


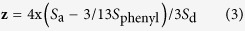



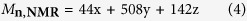


Thus, all these evidences strongly supported that PEG-*b*-PBPFCBPMA-*b*-P*t*BMA **3** triblock copolymers possessed well-defined structures with relatively low polydisperistis (*M*_w_/*M*_n_ ≤ 1.43): hydrophilic PEG block (45/113 repeated units) and hydrophobic PBPFCBPMA (54/69 repeated units) and P*t*BMA (9–95 repeated units).

### Conversion of PEG-*b*-PBPFCBPMA-*b*-P*t*BMA to PEG-*b*-PBPFCBPMA-*b*-PMAA

Although PEG-*b*-PBPFCBPMA-*b*-P*t*BMA **3** triblock copolymer is certainly a kind of amphiphilic copolymer due to the existence of hydrophilic PEG block and hydrophobic P*t*BMA and PBPFCBPMA segments, the amphiphilicity of the triblock copolymer can be further tuned by the transformation of pendant hydrophobic *tert*-butoxycarbonyls in P*t*BMA chains to hydrophilic carboxyls because that P*t*BMA has been widely reported to be entirely hydrolyzed into PMAA without affecting other functionalities^48^,^49^,^50^. Meanwhile, PMAA is a kind of polyacid whose solubility is dependent on pH value of solution. Therefore, the triblock copolymer bearing PMAA segment will be endowed with pH-responsiveness.

In the current case, *tert*-butoxycarbonyls of P*t*BMA block were hydrolyzed to carboxyls in CH_2_Cl_2_ at room temperature according to previous examples, in which excessive CF_3_COOH (20 folds) and long reaction time (24 h) were employed to guarantee the complete conversion of *tert*-butyoxycarbonyls. The product was washed with toluene, the solvent for PEG-*b*-PBPFCBPMA-Br **2** and PEG-*b*-PBPFCBPMA-*b*-P*t*BMA **3** and the precipitator for PEG-*b*-PBPFCBPMA-*b*-PMAA **4**, and no trace of PEG-*b*-PBPFCBPMA-Br **2** and PEG-*b*-PBPFCBPMA-*b*-P*t*BMA **3** was detected in the filtrate using GPC and ^1^H NMR. This observation also evidenced the complete initiation of PEG-*b*-PBPFCBPMA-Br **2** and conversion of PEG-*b*-PBPFCBPMA-*b*-P*t*BMA **3**. The molecular weights and molecular weight distributions (*M*_w_/*M*_n_ ≤ 1.41) of four hydrolyzed resultants are similar to those before hydrolysis as summarized in Table 4, which proved that polymeric skeleton was not affected during the acidic hydrolysis and the products kept well-defined structures.

**Figure 7:**
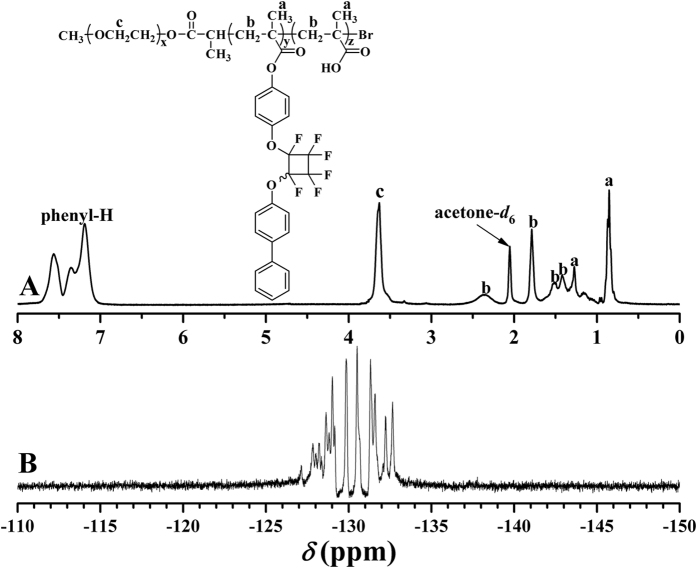
^1^H (**A**) and ^19^F (**B**) NMR spectra of PEG-*b*-PBPFCBPMA-*b*-PMAA **4** triblock copolymer in acetone-*d*_6_.

The chemical structure of the hydrolyzed product was examined by ^1^H NMR, ^19^F NMR, ^13^C NMR, and FT-IR, respectively. Figure 7A shows ^1^H NMR spectrum after the hydrolysis and it distinctly displayed the proton resonance signals originating from PEG and PBPFCBPMA blocks including the characteristic peaks at 3.63 (OC*H*_2_C*H*_2_) and 7.19–7.57 (phenyl) ppm. This evidence plus the typical multiplets (−127.0~−133.1 ppm) assigned to PFCB ring in ^19^F NMR spectrum (Fig. 7B) and the characteristic carbon resonance signals attributed to PEG and PBPFCBPMA segments in ^13^C NMR spectrum witnessed that PEG and PBPFCBPMA segments kept inert during the hydrolysis. The strong peak at 1.47 ppm corresponding to 9 protons of *tert-*butyls of P*t*BMA segment in Fig. 6 and the carbon resonance signals at 27.9 (C(*C*H_3_)_3_) and 80.5 (*C*(CH_3_)_3_) ppm in ^13^C NMR spectrum before the hydrolysis disappeared after the hydrolysis^52^, which indicated the complete hydrolysis of *tert*-butoxycarbonyls.

**Figure 8:**
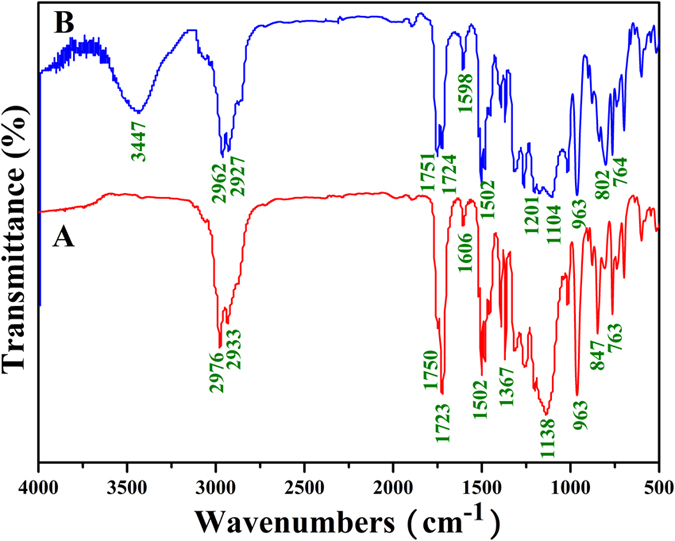
FT-IR spectra of PEG-*b*-PBPFCBPMA-*b*-P*t*BMA **3** (**A**) and PEG-*b*-PBPFCBPMA-*b*-PMAA **4** (**B**) triblock copolymers.

The formation of PMAA block was verified by FT-IR. It can be seen from Fig. 8A that a new broad peak attributed to the newly formed carboxyls appeared at 3447 cm^−1^ after the hydrolysis, which was absent before the hydrolysis as shown in Fig. 8B. Furthermore, typical signals corresponding to PEG (1104 cm^−1^) and PBPFCBPMA (1502, 963, and 764 cm^−1^) segments still appeared in FT-IR spectrum after the hydrolysis. Now, it is clear that PEG-*b*-PBPFCBPMA-*b*-P*t*BMA **3** triblock copolymer was selectively hydrolyzed into PEG-*b*-PBPFCBPMA-*b*-PMAA **4** triblock copolymer in acidic environment without affecting PEG and PBPFCBPMA segments.

### Self-assembly of PEG-*b*-PBPFCBPMA-*b*-PMAA amphiphilic triblock copolymer

**Figure 9:**
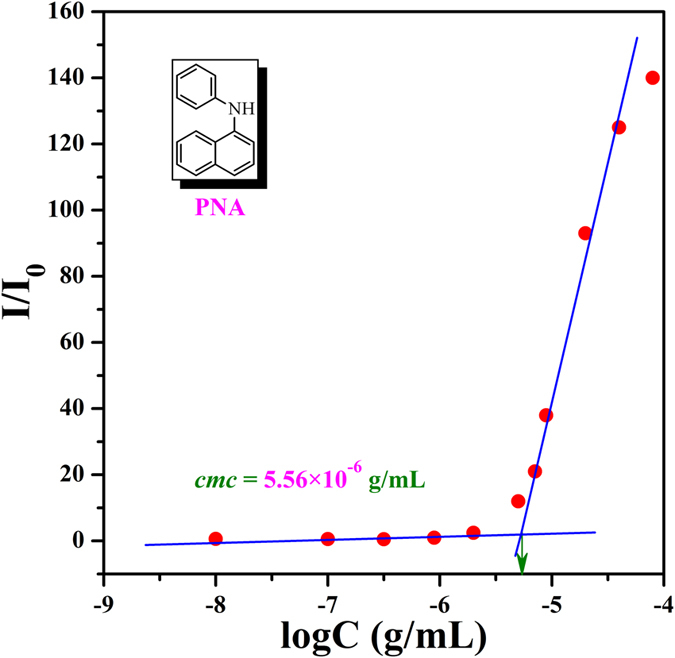
Dependence of fluorescence intensity ratio of PNA emission band at 418 nm on the concentration of PEG-*b*-PBPFCBPMA-*b*-PMAA **4a** ([PNA] = 10^−6 ^mol/L).

Similar to conventional small molecule surfactant, the amphiphilic nature of PEG-*b*-PBPFCBPMA-*b*-PMAA **4** triblock copolymers, consisting of hydrophilic PEG and PMAA, and hydrophobic semi-fluorinated PBPFCBPMA segments, can endow the copolymer with capability to self-assemble in aqueous media. Therefore, the triblock copolymer possesses a critical micellization concentration (*cmc*), indicative of the solubility (dispersibility) or amphiphilicity of amphiphilic copolymer. Herein, fluorescence spectroscopy was utilized to determine the *cmc* of PEG-*b*-PBPFCBPMA-*b*-PMAA **4** triblock copolymer in aqueous media using PNA as probe. As it is well-known, fluorescence spectrum of PNA is very sensitive to the environment and the polarity of its surrounding^53^. PNA can be solubilized within the interior of the hydrophobic part of micelles so that its fluorescence intensity will increase with the ascending of the concentration of amphiphilic copolymer^54^. When the concentration of copolymer **4a** was low, the variation of I/I_0_ was very small; however, I/I_0_ increased sharply when the concentration of copolymer **4a** exceeded a certain value, which meant PNA probe was incorporated into the hydrophobic region of micelles. Therefore, *cmc* of PEG-*b*-PBPFCBPMA-*b*-PMAA **4a** triblock copolymer was then determined to be the intersection of two straight lines with a value of 5.56 × 10^−6^ g/mL as shown in Fig. 9 and all *cmc*s of PEG-*b*-PBPFCBPMA-*b*-PMAA **4** triblock copolymers are summarized in Table 5.

Because PEG-*b*-PBPFCBPMA-*b*-PMAA **4** triblock copolymer possessed a pH-responsive PMAA segment, *cmc*s of all four triblock copolymers were measured in both neutral (pH = 7.0) and basic (pH = 10.0) aqueous solutions as listed in Table 5 and these values were certainly comparable with those of polymeric amphiphiles^55^. It should be noted that *cmc* of PEG-*b*-PBPFCBPMA-*b*-PMAA **4** triblock copolymer initiated by the same PEG-*b*-PBPFCBPMA-Br **2** diblock copolymer (**4a** and **4b** from **2a**, and **4c** and **4d** from **2b**) increased with the elevating of the block length of PMAA segment. Additionally, *cmc* of copolymer **4a** rose from 5.56 × 10^−6^ g/mL (pH = 7.0) to 6.23 × 10^−6^ g/mL (pH = 10.0) while the neutral aqueous solution turned basic, which can be attributed to the raised solubility of triblock copolymer in basic aqueous media since that PMAA segment behaved as an acid anion with a pH value of 10.0^56^.

**Figure 10:**
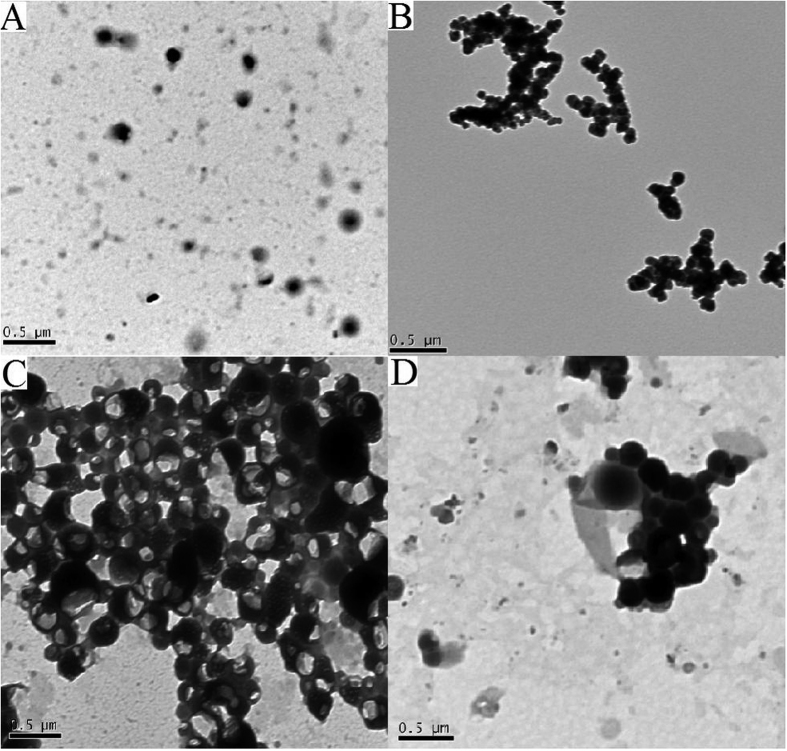
TEM images of micelles formed by PEG-*b*-PBPFCBPMA-*b*-PMAA **4** in neutral aqueous solution (pH = 7.0) with a polymer concentration of 1 mg/mL and an initial water content of 30 wt%, (**A**) **4a,** (**B**) **4b**, (**C**) **4c**, and (**D**) **4d**.

The self-assembly of PEG-*b*-PBPFCBPMA-*b*-PMAA **4** amphiphilic triblock copolymer in neutral aqueous solution was investigated by TEM and the micellar solution was prepared by dialysis approach, which is a frequently used method to prepare micelles formed by amphiphilic block copolymer^57^. It can be clearly seen from Fig. 10 that spherical micelles were formed for all four PEG-*b*-PBPFCBPMA-*b*-PMAA **4** triblock copolymers with average diameters of 150–300 nm in neutral aqueous solution. Hydrodynamic diameter (*D*_h_) of these micelles obtained from dynamic light scattering (DLS) were 181, 166, 324, and 307 nm for copolymer **4a**, **4b**, **4c**, and **4d**, respectively, which were all higher than those obtained from TEM due to the hydration effect. However, most of micelles were much larger than the theoretical length of triblock copolymer, which inferred that large compound micelles, rather than common spherical micelles, were formed^58^,^59^. For these large compound micelles in the current case, micellar structure can be deemed that hydrophilic PEG and PMAA segments formed the corona of micelles and the core was consisted of numerous reverse micelles with islands of PEG and PMAA segments in continuous phase of hydrophobic PBPFCBPMA segments according to previous literatures^29^,^58^. One can notice that copolymer **4c** formed bowl-shaped micelles (Fig. 10C), which is a special large compound spherical micelle containing an asymmetrically placed single void space breaking through the surface^60^. Previous reports suggested that the internal viscosity of micelles in the course of formation of micelle with the removal of good solvent from the micelle solution was highly important for the formation of bowl-shaped micelles and the viscosity window for the formation of bowl-shaped micelle was very narrow^60^,^61^,^62^. In the present work, the relatively rigid PFCB aryl ether side groups in PBPFCBPMA segments combining with the relatively suitable fluorine-containing block length may make the internal viscosity of part of micelles be in the viscosity window suitable for forming bowl- shaped micelles. Thus, both large compound micelles and bowl-shaped micelles were observed for copolymer **4** in neutral environment.

### Encapsulation of hydrophobic and hydrophilic agents by large compound micelle

The ability of common spherical micelle to package or transport hydrophobic compounds in its hydrophobic core has been widely employed as delivery vehicles for pharmaceuticals, gene therapy agents, pesticides, and personal care products^63^. However, in many cases one might wish to encapsulate both hydrophobic and hydrophilic compounds simultaneously, that is to say, we need a multi-compartment micelle to deliver two different incompatible compounds to the same place at the same time. Although it was well recognized that for large compound micelle, hydrophilic block of amphiphilic copolymer forms the corona of micelle and the core consists of numerous reverse micelles with hydrophilic islands in continuous hydrophobic phase, the encapsulating ability of large compound micelle formed by fluoropolymers for hydrophobic and hydrophilic agents has not been examined yet^58^.

**Figure 11:**
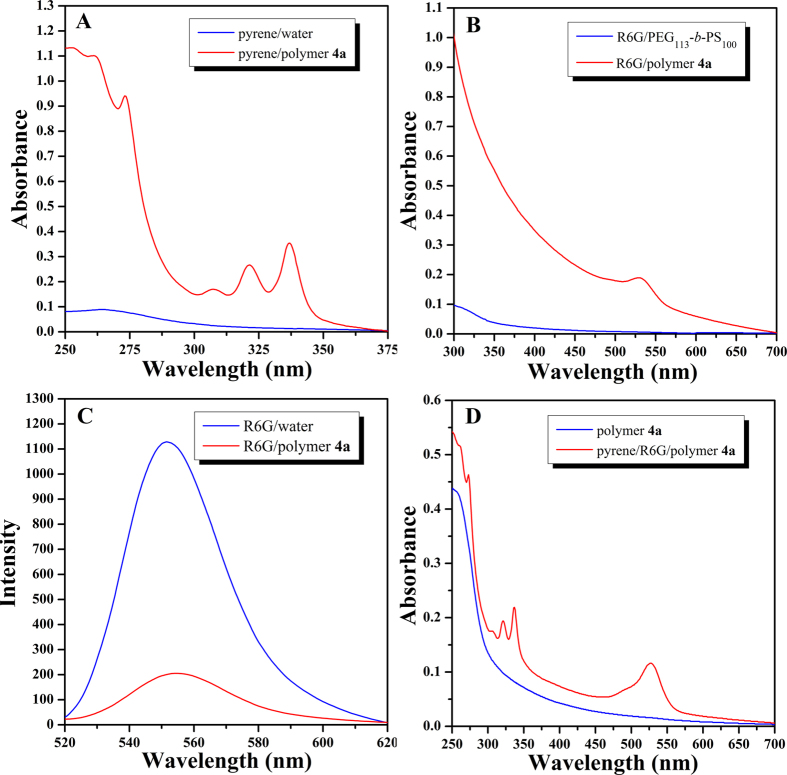
(**A**) UV/vis absorption spectra of pyrene in water and aqueous micellar solution of PEG-*b*-PBPFCBPMA-*b*-PMAA **4a** triblock copolymer. (**B**) UV/vis absorption spectra of R6G in aqueous micellar solutions of PEG-*b*-PBPFCBPMA-*b*-PMAA **4a** triblock copolymer and PEG_113_-*b*-PS_100_ diblock copolymer. (**C**) Fluorescence emission spectra of R6G in water and aqueous micellar solution of PEG-*b*-PBPFCBPMA-*b*-PMAA **4a** triblock copolymer. (**D**) UV/vis absorption spectra of pyrene and R6G in aqueous micellar solution of PEG-*b*-PBPFCBPMA-*b*-PMAA **4a** triblock copolymer, and the micellar solution of copolymer **4a** for control experiment.

With the fact that all four PEG-*b*-PBPFCBPMA-*b*-PMAA **4** triblock copolymers formed large compound micelles in aqueous media, we hypothesize the hydrophilic and hydrophobic domains within the core of large compound micelles might be able to encapsulate both hydrophobic and hydrophilic agents. To demonstrate this hypothesis, pyrene, which can dissolve in hydrophobic domain, and Rhodamine 6G (R6G), which can selectively dissolve in hydrophilic domain, were used as model guest molecules to measured encapsulating ability of large compound micelles formed by PEG-*b*-PBPFCBPMA-*b*-PMAA **4a** triblock copolymer. We firstly performed the encapsulation of hydrophobic dye of pyrene in aqueous micellar solution of copolymer **4a** for probing the nature of micelles. The loading content of pyrene, 16.6 μg pyrene/1 mg micelle, was determined by UV/vis absorption spectroscopy using standard curve at 337 nm (see Fig. S1A in Supporting Information). The solubility of pyrene in water is very poor so that the aqueous solution of pyrene (after filtration to remove insoluble pyrene) showed almost no UV absorption of pyrene (Fig. 11A). However, we can clearly see the typical UV absorption of pyrene at 337 nm (Fig. 11A) in aqueous micellar solution of copolymer **4a**, which indicated that micelles are able to sequester pyrene from water and solubilize pyrene within the hydrophobic domain^63^,^64^.

We then performed the encapsulation of hydrophilic dye of R6G in aqueous micellar solution of copolymer **4a**. The loading content of R6G, 6.77 μg R6G/1 mg micelle, was also determined by UV/vis absorption spectroscopy using standard curve at 529 nm (see Fig. S1B in Supporting Information). Figure 11B shows UV absorption spectrum of the micellar solution of copolymer **4a** containing R6G (after dialysis to remove water-soluble R6G) and a typical absorption peak of R6G appeared at 529 nm, which was absent in that of the aqueous micellar solution of PEG_113_-*b*-PS_100_ (common spherical micelle, see Fig. S2 in Supporting Information) containing R6G (prepared with same procedure) as shown in Fig. 11B. This fact clearly proved that large compound micelles formed by copolymer **4a** can sequester R6G from water and solubilize R6G within the hydrophilic domain^64^,^65^, while the usual spherical micelles formed by PEG_113_-*b*-PS_100_ amphiphilic diblock copolymer can not store R6G^64^. Moreover, as R6G exhibits self-quenching property, it is possible to investigate whether R6G is really sequestered inside the micelles. If a certain amount of R6G is uniformly distributed in pure water, there will be no self-quenching effect. Nevertheless, when R6G is distributed in aqueous micellar solution, the local concentration of R6G within the micelles increases dramatically, which would result in self-quenching although the concentration of R6G in micelles is same as that of pure aqueous solution^64^,^65^. The same micellar solution of copolymer **4a** containing R6G for UV measurement was employed for fluorescence spectroscopy measurement in comparison with the control experiment of pure aqueous solution of R6G with same concentration (same UV absorbance, Fig. S1). It can be distinctly seen from Fig. 11C that the fluorescence intensity of micellar solution of copolymer **4a** containing R6G was just 18% of that of pure aqueous solution of R6G with same concentration, this clearly indicating the obvious self-quenching. This self-quenching fluorescence of R6G further demonstrated that hydrophilic R6G is actually sequestered inside the micelles formed by copolymer **4a**.

**Figure 12:**
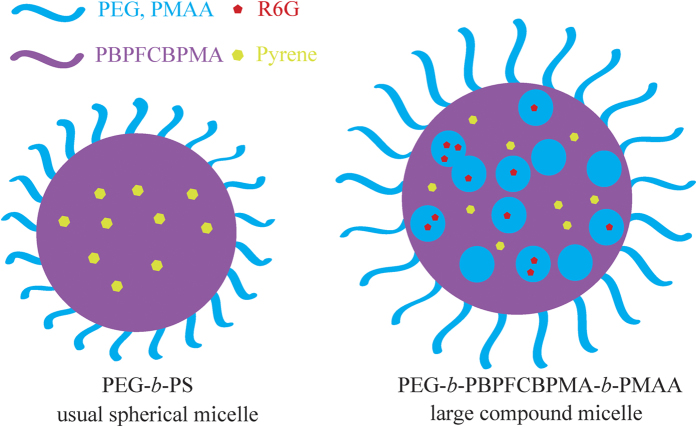
Schematic illustration of encapsulating capacities of usual spherical micelle formed by PEG_113_-*b*-PS_100_ diblock copolymer and large compound micelle formed by PEG-*b*-PBPFCBPMA-*b*-PMAA triblock polymer for hydrophobic and hydrophilic compounds by employing R6G and pyrene as model hydrophilic and hydrophobic agents, respectively.

Finally, both pyrene and R6G were co-encapsulated in aqueous micellar solution of copolymer **4a**. Two typical UV absorption peaks appeared at 337 (pyrene) and 529 (R6G) nm in UV/vis absorption spectrum (Fig. 11D), which was absent in UV spectrum of aqueous micellar solution of copolymer **4a** (Fig. 11D), which clearly confirmed that both hydrophilic R6G and hydrophobic pyrene model loading agents were solubilized within different domains of large compound micelles. All above-mentioned results evidenced that large compound micelles formed by PEG-*b*-PBPFCBPMA-*b*-PMAA **4** semi-fluorinated amphiphilic triblock copolymers can separately or simultaneously uptake hydrophobic and hydrophilic model loading agents (Fig. 12), which are certainly potential delivery vehicles.

## Methods

### Materials

*tert*-Butyl methacrylate (*t*BMA, Aldrich, 99%) was distilled with CaH_2_ under reduced pressure prior to use. Copper(I) bromide (CuBr, Aldrich, 98%) was purified by stirring overnight over CH_3_COOH at room temperature, followed by washing with ethanol, diethyl ether, and acetone prior to drying at 40 °C *in vacuo* for one day. *N*-Phenyl-1-naphthylamine (PNA, Alfa Aesar, 97%) was purified by recrystallization in ethanol three times. 2-Butanone (Aldrich, 99%) was dried with CaCl_2_ and then distilled under vacuum. Dichloromethane (CH_2_Cl_2_, Aldrich, 99.5%) was dried over KOH and distilled from CaH_2_ under N_2_ prior to use. Poly(ethylene glycol) methyl ether (PEG-OH, *M*_n_ = 2,000 and 5,000 g/mol, Aldrich), 2-bromopropionyl bromide (Alfa Aesar, 97%), *N*,*N*,*N*′,*N*′,*N*″-pentamethyl diethylenetriamine (PMDETA, Aldrich, 99%), 4-(dimethylamino)pyridine (DMAP, Aldrich, 99%), triethylamine (TEA), trifluoroacetic acid (TFA, Aldrich, 99%), and pyrene (Aldrich, 99%), rhodamine 6 G (R6G, Aldrich, 99%) were used as received. Other solvents were obtained from commercial sources and used as received. *p*-(2-(4-Biphenyl)perfluorocyclobutoxy)phenyl methacrylate (BPFCBPMA)^23^ and PEG_113_-*b*-PS_100_ diblock copolymer^66^ was synthesized according to previous report.

### Measurements

All NMR analyses were performed on a Bruker Avance 500 spectrometer (500 MHz) in CDCl_3_ or acetone-*d*_6_; tetramethylsilane (^1^H NMR) and CDCl_3_ (^13^C NMR) were used as internal standards, and CF_3_CO_2_H was used as external standard for ^19^F NMR. FT-IR spectra were recorded on a Nicolet AVATAR-360 spectrophotometer with a 4 cm^−1^ resolution. Relative molecular weights and molecular weight distributions were measured by conventional gel permeation chromatography (GPC) system equipped with a Waters 1515 Isocratic HPLC pump, a Waters 2414 refractive index detector, and a set of Waters Styragel columns (HR3 (500–30,000), HR4 (5,000–600,000) and HR5 (50,000–4,000,000), 7.8 × 300 mm, particle size: 5 μm). GPC measurements were carried out at 35 °C using THF as eluent with a flow rate of 1.0 mL/min. The system was calibrated with linear poly(ethylene glycol) standards (PEG-Br macroinitiators) and poly(methyl methacrylate) standards (PEG-*b*- PBPFCBPMA-Br, PEG-*b*-PBPFCBPMA-*b*-P*t*BMA, and PEG-*b*-PBPFCBPMA-*b*-PMAA block copolymers). UV/vis absorption spectra were measured by a Hitachi U-2910 spectrophotometer with a rate of 200 nm/min. Steady-state fluorescence spectra were measured at 20 °C on a Hitachi F-2700 spectrophotometer with the band width of 5 nm for excitation and emission, the emission intensity at 418 nm (λ_ex_ = 340 nm) was recorded to determine the critical micelle concentration (*cmc*). With the band width of 2.5 nm for excitation and emission, the emission intensity of R6G at 551 nm (λ_ex_ = 500 nm) was recorded. Hydrodynamic diameter (*D*_h_) was measured by dynamic light scattering (DLS) with a Malvern Nano-ZS90 Zetasizer, the sample was allowed to equilibrate for 2 min prior to the measurement. TEM images were obtained by a JEOL JEM-1230 instrument operated at 80 kV.

### End functionalization of PEG-OH

In a typical procedure, PEG-OH (*M*_n_ = 2,000 g/mol, 3.000 g, 1.5 mmol −OH), DMAP (0.549 g, 4.5 mmol), TEA (0.622 mL, 4.5 mmol), and CH_2_Cl_2_ (20 mL) were added to a 200 mL Schlenk flask (flame-dried under vacuum prior to use) sealed with a rubber septum for degassing and kept under N_2_. Next, 2-bromopropionyl bromide (1.57 mL, 15.0 mmol) in 10 mL of CH_2_Cl_2_ was added via a gastight syringe at 0 °C. The reaction proceeded at 25 °C for 24 h. The solvent was rotary evaporated after filtration. The crude product was dissolved in *n*-hexane and washed with brine, and the organic layer was dried over MgSO_4_ overnight. After evaporation of the solvent, the residue was purified by column chromatography using CH_2_Cl_2_/methanol (30:1) as eluent to give PEG-Br **1a** (1.14 g, 38.1%) as white powder. GPC: *M*_n_ = 2,200 g/mol, *M*_w_/*M*_n_ = 1.09. FT-IR (KBr): *ν* (cm^−1^): 2870, 2359, 2336, 1741, 1702, 1688, 1458, 1349, 1297, 1249, 1205, 1107, 1023, 999, 950, 844. ^1^H NMR (CDCl_3_): *δ* (ppm): 1.84 (3H, COCH(C*H*_3_)Br), 3.38 (3H, OC*H*_3_), 3.65 (4H, OC*H*_2_C*H*_2_), 4.33 (2H, CH_2_C*H*_2_O_2_C), 4.42 (1 H, COC*H*(CH_3_)Br). For PEG-Br **1b** obtained from PEG-OH (*M*_n_ = 5,000 g/mol): GPC: *M*_n_ = 5,300 g/mol, *M*_w_/*M*_n_ = 1.13.

### ATRP block copolymerization of BPFCBPMA

In a typical procedure, CuBr (1.5 mg, 0.01 mmol) and PEG-Br **1a** (*M*_n_ = 2,200 g/mol, *M*_w_/*M*_n_ = 1.09, 20.0 mg, 0.01 mmol ATRP initiating group) were first added to a 10 mL Schlenk flask (flame-dried under vacuum prior to use) sealed with a rubber septum for degassing and kept under N_2_. Next, BPFCBPMA (0.3071 g, 0.60 mmol), 2-butanone (0.5 mL), and PMDETA (2.1 μL, 0.01 mmol) were added via a gastight syringe. The flask was degassed by three cycles of freezing-pumping-thawing followed by immersing the flask into an oil bath set at 70 °C. The polymerization was terminated by immersing the flask into liquid N_2_ after 12 h. The mixture was diluted by THF and passed through an alumina column to remove the residual copper catalyst. The solution was concentrated and precipitated into cold *n*-hexane. The solid was washed with methanol, the precipitator for PEG-*b*-PBPFCBPMA-Br diblock copolymer, to remove any possible unreacted PEG-Br macroinitiator and no trace of PEG-Br was detected in the filtrate using GPC and ^1^H NMR. After repeated purification by dissolving in THF and precipitating in *n*-hexane, 168.7 mg of PEG-*b*-PBPFCBPMA-Br **2a** was obtained as a white powder after drying *in vacuo* overnight. GPC: *M*_n_ = 14,900 g/mol, *M*_w_/*M*_n_ = 1.43. FT-IR (KBr): *ν* (cm^−1^): 2869, 1751, 1502, 1314, 1201, 1104, 962. ^1^H NMR (CDCl_3_): *δ* (ppm): 0.87, 1.35 (3H, CH_2_CC*H*_3_), 1.44, 2.08 (2H, C*H*_2_CCH_3_), 3.65 (4H, OC*H*_2_C*H*_2_), 6.98–7.44 (13H, phenyl). ^19^F NMR (CDCl_3_): *δ* (ppm): −127.0~−132.8 (6F, cyclobutyl-*F*_6_). ^13^C NMR (CDCl_3_): *δ* (ppm): 20.5, 30.3, 45.3, 68.9, 70.6, 105.0, 119.5, 138.3, 146.3, 152.9, 175.6.

### ATRP block copolymerization of *t*BMA

In a typical procedure, PEG-*b*-PBPFCBPMA-Br **2a** (*M*_n,GPC_ = 14,900 g/mol, *M*_w_/*M*_n_ = 1.43, *M*_n,NMR_ = 29,600 g/mol, 59.2 mg, 0.002 mmol initiating group) and CuBr (0.287 mg, 0.002 mmol) were first added to a 10 mL Schlenk flask (flame-dried under vacuum prior to use) sealed with a rubber septum for degassing and kept under N_2_. Next, *t*BMA (0.016 mL, 0.1 mmol), 2-butanone (0.5 mL), and PMDETA (0.4 μL, 0.002 mmol) were added via a gastight syringe. The flask was degassed by three cycles of freezing-pumping-thawing followed by immersing the flask into an oil bath set at 70 °C. The polymerization was terminated by immersing the flask into liquid N_2_ after 12 h. The mixture was diluted by THF and passed through an alumina column to remove the residual copper catalyst. The solution was concentrated and precipitated into cold *n*-hexane. After repeated purification by dissolving in THF and precipitating in *n*-hexane, 68.0 mg of PEG-*b*-PBPFCBPMA-*b*-P*t*BMA **3a** was obtained as a white powder after drying *in vacuo* overnight. GPC: *M*_n_ = 17,100 g/mol, *M*_w_/*M*_n_ = 1.40. FT-IR (KBr): *ν* (cm^−1^): 2976, 2933, 1750, 1724, 1606, 1515, 1502, 1478, 1456, 1367, 1318, 1393, 1202, 1175, 1138, 1015, 963, 847, 763. ^1^H NMR (CDCl_3_): *δ* (ppm): 1.09, 1.18 (3H, CH_2_CC*H*_3_), 1.47 (9H, C(C*H*_3_)_3_ and 2H, C*H*_2_CCH_3_), 1.88, 2.35 (2H, C*H*_2_CCH_3_), 3.58 (4H, OC*H*_2_C*H*_2_), 7.18–7.56 (13H, phenyl). ^19^F NMR (CDCl_3_): *δ* (ppm): −127.8~−132.1 (m, cyclobutyl-*F*_6_). ^13^C NMR (CDCl_3_): *δ* (ppm): 20.6, 27.9, 30.4, 45.6, 69.1, 70.4, 80.5, 105.2, 119.2, 138.6, 146.2, 153.2, 175.3.

### Selective acidic hydrolysis of PEG-*b*-PBPFCBPMA-*b*-P*t*BMA

In a typical procedure, PEG-*b*-PBPFCBPMA-*b*-P*t*BMA **3a** (*M*_n,GPC_ = 17,100 g/mol, *M*_w_/*M*_n_ = 1.40, *M*_n,NMR_ = 31,700 g/mol, 50.0 mg, 0.024 mmol *tert*-butyoxycarbonyl) and CH_2_Cl_2_ (3.0 mL) were added to a 50 mL three-neck flask. The solution was stirred at 0 °C for 30 min followed by adding TFA (35.6 μL, 0.48 mmol) and the reaction mixture was warmed to 25 °C. After stirring at room temperature for 24 h, the solution was concentrated and precipitated into cold *n*-hexane. After filtration, 25.2 mg of white powder, PEG-*b*-PBPFCBPMA-*b*-PMAA **4a**, was obtained after drying *in vacuo* overnight. The product was also washed with toluene, the solvent for PEG-*b*- PBPFCBPMA-Br and PEG-*b*-PBPFCBPMA-*b*-P*t*BMA and the precipitator for PEG-*b*-PBPFCBPMA-*b*-PMAA, and no trace of PEG-*b*-PBPFCBPMA-Br and PEG-*b*-PBPFCBPMA-*b*-P*t*BMA was detected in the filtrate using GPC and ^1^H NMR. GPC: *M*_n_ = 16,900 g/mol, *M*_w_/*M*_n_ = 1.37. FT-IR (KBr): *ν* (cm^−1^): 3447, 2962, 2927, 1751, 1724, 1598, 1502, 1483, 1456, 1317, 1263, 1201, 1104, 1018, 963, 802, 764. ^1^H NMR (acetone-*d*_6_): *δ* (ppm): 0.88, 1.28 (3H, CH_2_CC*H*_3_), 1.43, 1.52 1.79, 2.36 (2H, C*H*_2_CCH_3_), 3.63 (4H, OC*H*_2_C*H*_2_), 7.19–7.57 (13H, phenyl). ^19^F NMR (acetone-*d*_6_): *δ* (ppm): −127.0~−133.1 (m, cyclobutyl-*F*_6_). ^13^C NMR (acetone-*d*_6_): *δ* (ppm): 20.8, 30.1, 45.5, 69.2, 70.5, 105.2, 119.2, 138.1, 146.6, 152.7, 175.4.

### Determination of critical micelle concentration

PNA was used as fluorescence probe to measure the critical micelle concentration (*cmc*) of PEG-*b*-PBPFCBPMA-*b*-PMAA **4** triblock copolymer in aqueous media. Acetone solution of PNA ([PNA] = 2 mM) was added to a large amount of water until the concentration of PNA reached 0.001 mM. The solutions for fluorescence measurement were obtained by adding different amounts of THF solutions of PEG-*b*-PBPFCBPMA-*b*-PMAA **4** triblock copolymer (1, 0.1, 0.01, or 0.001 mg/mL) to water containing PNA ([PNA] = 0.001 mM).

### Micellar morphology

THF solution of PEG-*b*-PBPFCBPMA-*b*-PMAA **4** triblock copolymer (1.0 mg/mL) was first filtered through a membrane with a nominal pore size of 0.45 μm. Next, a certain amount of deionized water was added slowly (0.36 mL/h) to 1.00 g of THF solution of triblock copolymer **4** by a microsyringe until the preset water content (30 wt%) was reached. Subsequently, the solution was dialyzed against deionized water with slow stirring for 3 days to remove THF completely, and deionized water was changed twice a day to obtain aqueous micellar solution. For TEM studies, 10 μL of micellar solution was deposited on an electron microscopy copper grid coated with carbon film and the water evaporated at room temperature.

### Encapsulation of hydrophobic pyrene in micelles

THF solution of PEG-*b*-PBPFCBPMA-*b*-PMAA **4** triblock copolymer and pyrene was first filtered through a membrane with a nominal pore size of 0.45 μm. Next, a certain amount of deionized water was added slowly (0.36 mL/h) to 0.6 mL of THF solution of copolymer **4** and pyrene by a microsyringe until the concentrations of polymer and pyrene reached 0.08 mg/mL and 0.02 mmol/L, respectively. After stirring at room temperature for 1 h, the solution was dialyzed against deionized water with slow stirring for 3 days. For the control experiment, only pyrene was added to deionized water. Both solutions were filtered through a 0.45 μm syringe filter and the obtained solutions were employed for the measurement of UV absorption spectroscopy.

### Encapsulation of hydrophilic Rhodamine 6 G in micelles

A certain amount of deionized water was added slowly (0.36 mL/h) to the mixed solution of copolymer **4** (0.4 mL, THF) and Rhodamine 6 G (0.2 mL, H_2_O) by a microsyringe until the concentrations of polymer and R6G reached 0.08 mg/mL and 0.02 mmol/L, respectively. After stirring at room temperature for 1 h, the solution was dialyzed against deionized water with slow stirring for 3 days until the dialysate did not show any detectable UV signal. The obtained solution was used for the measurements of UV absorption and fluorescence spectroscopy. Similar procedure was used for PEG_113_-*b*-PS_100_ diblock copolymer.

### Co-encapsulation of Rhodamine 6 G and pyrene in micelles

A certain amount of deionized water was added slowly (0.36 mL/h) to the mixed solution of copolymer **4** (0.4 mL, THF), pyrene (0.2 mL, THF) and Rhodamine 6 G (0.2 mL, H_2_O) by a microsyringe until the concentrations of polymer, pyrene and R6G reached 0.08 mg/mL, 0.02 mmol/L and 0.02 mmol/L, respectively. After stirring at room temperature for 1 h, the solution was dialyzed against deionized water with slow stirring for 3 days until the dialysate did not show any detectable UV signal. The obtained solution was used for the measurement of UV absorption spectroscopy.

## Conclusions

We have presented a convenient synthesis of well-defined PFCB-containing PEG-*b*-PBPFCBPMA-*b*-P*t*BMA amphiphilic ABC triblock copolymers with relatively narrow molecular weight distributions (*M*_w_/*M*_n_ ≤ 1.43) via sequential ATRP. With the successfully preparation of PEG-Br macroinitiator, the versatility of ATRP can make PBPFCBPMA and P*t*BMA segments substituted by other polymeric chains which obviously paves a convenient way for developing new PFCB aryl ether-based amphiphilic block copolymers. More significantly, the amphiphilicity of PEG-*b*-PBPFCBPMA-*b*-P*t*BMA triblock copolymer could be further adjusted by hydrolyzing pendant *tert*-butoxycarbonyls in P*t*BMA block to carboxyls while this transformation endowed the resulting PEG-*b*-PBPFCBPMA-*b*-PMAA triblock copolymer with pH-responsiveness, which possesses a higher *cmc* in basic surrounding in comparison with that in neutral environment. The self-assembly behavior of PEG-*b*-PBPFCBPMA-*b*-MAA amphiphilic triblock copolymer in aqueous media was investigated by TEM, and large compound micelles and bowl-shaped micelles were formed in neutral aqueous solution. The relatively rigid structure of PFCB aryl ether side groups and the relatively suitable length of fluorine-containing block play important roles in self-assembly process. This observation can clearly not only show the importance of property of backbone of triblock copolymer on its self-assembled behavior, but deepen our understanding on the structure-property relationship of triblock copolymer. In particular, the multi-component structure of large compound micelles formed by PEG-*b*-PBPFCBPMA-*b*-PMAA triblock copolymer could be used as a multi-compartment delivery vehicle for the separated or simultaneous uptake of hydrophobic, hydrophilic compounds, which was verified to be able to separately or simultaneously package pyrene and R6G compounds in its different nanodomains.

## Additional Information

**How to cite this article**: Xu, B. *et al*. Perfluorocyclobutyl Aryl Ether-Based ABC Amphiphilic Triblock Copolymer. *Sci. Rep.* **6**, 39504; doi: 10.1038/srep39504 (2016).

**Publisher's note:** Springer Nature remains neutral with regard to jurisdictional claims in published maps and institutional affiliations.

## Supplementary Material

Supplementary Information

## Figures and Tables

**Figure 1:**
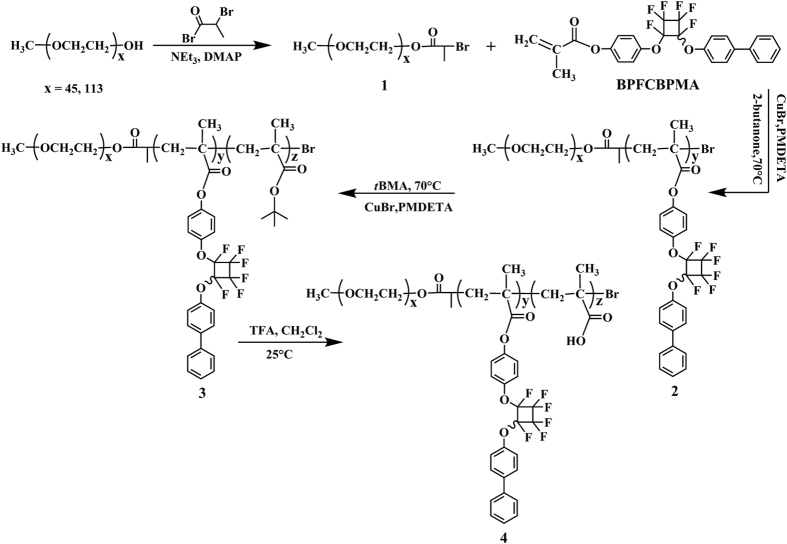
Synthesis of PEG-*b*-PBPFCBPMA-*b*-PMAA triblock copolymer by successive ATRP.

**Table 1:** Preparation of PEG-Br **1** macroinitiator^a^.

Entry	*M*_n,PEG-OH_ (g/mol)	x^d^	Time (h)	*M*_n,GPC_^e^ (g/mol)	*M*_w_/*M*_n_^e^
1a^b^	2,000	45	24	2,200	1.09
1b^c^	5,000	113	36	5,300	1.13

^a^Reaction temperature: 25 °C, [PEG-OH]:[TEA]:[DMAP] = 1:1:1, solvent: CH_2_Cl_2_.

^b^[2-bromopropionyl bromide]:[PEG-OH] = 10:1.

^c^[2-bromopropionyl bromide]: [PEG-OH] = 15:1.

^d^The number of EG repeated unit per chain.

^e^Measured by GPC in THF at 35 °C.

**Table 2:** Synthesis of PEG-*b*-PBPFCBPMA-Br **2** diblock copolymer^a^.

Entry	[BPFCBPMA]:[**1**]	*M*_n,GPC_^d^ (g/mol)	*M*_w_/*M*_n_^d^	*M*_n,NMR_^e^ (g/mol)	x-*b*-y^f^
2a^b^	60:1	14,900	1.43	29,600	45-*b*-54
2b^c^	80:1	24,200	1.45	40,200	113-*b*-69

^a^Polymerization temperature: 70 °C, polymerization time:12 h, [PEG-Br **1**]:[CuBr]: [PMDETA] = 1:1:1, solvent: 2-butanone.

^b^Initiated by PEG-Br **1a** (*M*_n_ = 2,200 g/mol, *M*_w_/*M*_n_ = 1.09).

^c^Initiated by PEG-Br **1b** (*M*_n_ = 5,300 g/mol, *M*_w_/*M*_n_ = 1.13).

^d^Measured by GPC in THF at 35 °C.

^e^Obtained from ^1^H NMR.

^f^The composition of diblock copolymer obtained from ^1^H NMR.

**Table 3:** Synthesis of PEG-*b*-PBPFCBPMA-*b*-P*t*BMA **3** triblock copolymer^a^.

Entry	[*t*BMA]:[**2**]	*M*_n,GPC_^d^ (g/mol)	*M*_w_/*M*_n_^d^	*M*_n,NMR_^e^ (g/mol)	x-y-z^f^
3a^b^	50:1	17,100	1.40	31,700	45-54-15
3b^b^	200:1	19,300	1.36	43,100	45-54-95
3c^c^	50:1	26,800	1.43	41,500	113-69-9
3d^c^	200:1	32,300	1.38	49,600	113-69-66

^a^[PEG-*b*-PBPFCBPMA-Br **2**]:[CuBr]:[PMDETA] = 1:1:1, solvent: 2-butanone, polymerization temperature: 70 °C, polymerization time: 12 h.

^b^Initiated by PEG-*b*-PBPFCBPMA-Br **2a** (*M*_n,GPC_ = 14,900 g/mol, *M*_w_/*M*_n_ = 1.43, *M*_n,NMR_ = 29,600 g/mol).

^c^Initiated by PEG-*b*-PBPFCBPMA-Br **2b** (*M*_n,GPC_ = 24,200 g/mol, *M*_w_/*M*_n_ = 1.45, *M*_n,NMR_ = 40,200 g/mol).

^d^Measured by GPC in THF at 35 °C.

^e^Obtained from ^1^H NMR.

^f^The composition of triblock copolymer obtained from ^1^H NMR.

**Table 4:** Synthesis of PEG-*b*-PBPFCBPMA-*b*-PMAA **4** triblock copolymer.

Entry	Starting material	*M*_n_^a^ (g/mol)	*M*_w_/*M*_n_^a^
4a	**3a**	16,900	1.37
4b	**3b**	17,700	1.38
4c	**3c**	25,700	1.41
4d	**3d**	30,200	1.36

^a^Measured by GPC in THF at 35 °C.

**Table 5:** *cmc* and micellar size of PEG-*b*-PBPFCBPMA-*b*-PMAA **4** triblock polymer.

Entry	*cmc*_pH_ = _7_^a^ (g/mL)	*cmc*_pH_ = _10_^a^ (g/mL)	*D*_h_^b^ (nm)	PDI^b^
4a	5.56 × 10^−6^	6.23 × 10^−6^	181	0.228
4b	5.90 × 10^−6^	6.83 × 10^−6^	166	0.194
4c	6.52 × 10^−6^	6.98 × 10^−6^	324	0.256
4d	7.07 × 10^−6^	7.81 × 10^−6^	307	0.261

^a^Determined by fluorescence spectroscopy using PNA as probe.

^b^Measured by dynamic light scattering (DLS).
