# Supplementary material for: Perfluorocyclobutyl Aryl Ether-Based ABC Amphiphilic Triblock Copolymer
Source: Sci Rep. 2016 Dec 21;6:39504. doi: 10.1038/srep39504 (PMC5175170; doi:10.1038/srep39504)
Supplement: Supplementary Information [file srep39504-s1.doc]

**Supporting Information for**

**Perfluorocyclobutyl Aryl Ether-Based ABC Amphiphilic Triblock Copolymer**

*Binbin Xu*,^#^ *Wenqiang Yao*,^#^ *Yongjun Li*,* *Sen Zhang*, *Xiaoyu Huang**

Key Laboratory of Synthetic and Self-Assembly Chemistry for Organic Functional Molecules, Shanghai Institute of Organic Chemistry, Chinese Academy of Sciences, 345 Lingling Road, Shanghai 200032, People’s Republic of China


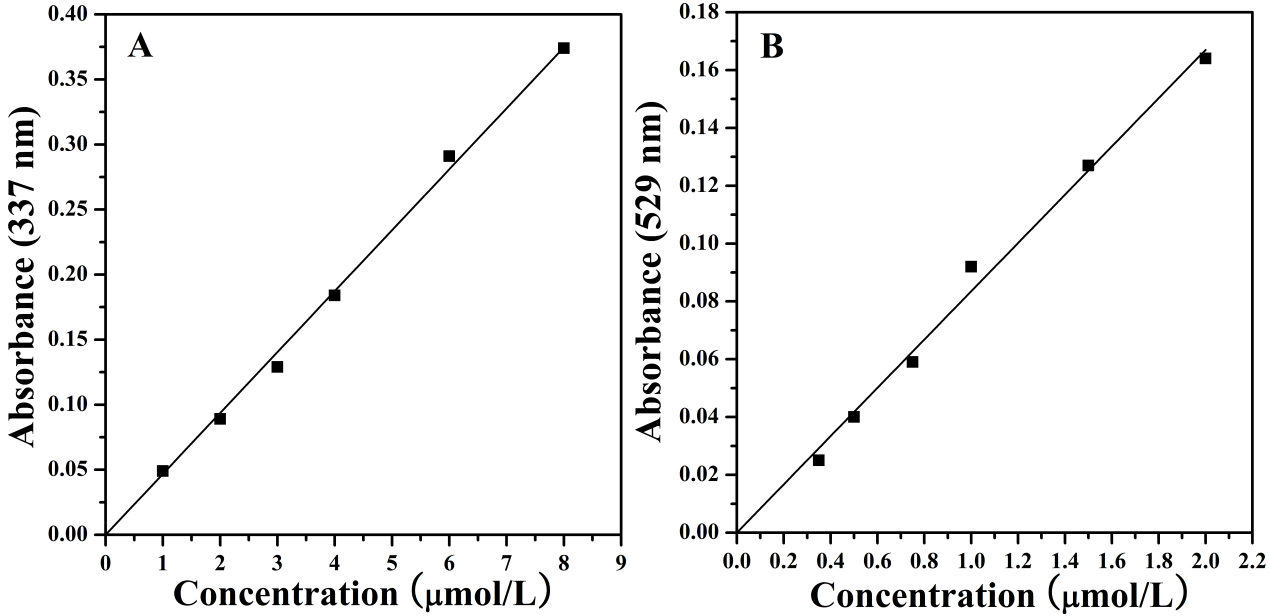


**Figure S1.** (A) UV-vis absorbance standard curve of pyrene at 337 nm, A = 0.046C. (B) UV-vis absorbance standard curve of R6G at 529 nm, A = 0.084C.


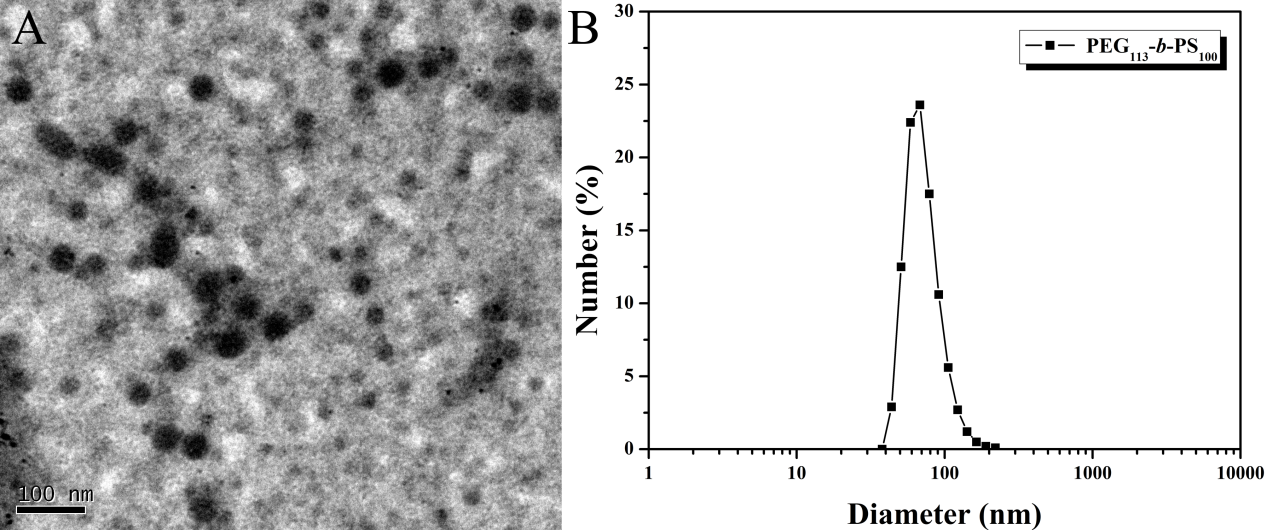


**Figure S2.** (A) TEM image and (B) diameter distribution of spherical micelles formed by PEG_113_-*b*-PS_100_ diblock copolymer.
